# Exhausted T cell phenotypes in disseminated coccidioidomycosis

**DOI:** 10.1172/jci.insight.203270

**Published:** 2026-04-20

**Authors:** Gregory D. Whitehill, Alexis V. Stephens, Timothy J. Thauland, Miguel A. Moreno Lastre, Matthew M. Tate, Sinem Beyhan, Royce H. Johnson, George R. Thompson, Maria Garcia-Lloret, Manish J. Butte

**Affiliations:** 1 Department of Medicine, Division of Infectious Diseases,; 2 Department of Human Genetics, and; 3 Department of Pediatrics, Division of Immunology, Allergy, and Rheumatology, UCLA, Los Angeles, California, USA.; 4 Veterans Affairs San Diego Healthcare System, Research Service, San Diego, California, USA.; 5 Department of Medicine, UCSD, La Jolla, California, USA.; 6 Department of Medicine, Division of Infectious Diseases, and; 7 Valley Fever Institute at Kern Medical Center, Bakersfield, California, USA.; 8 Department of Medicine, Division of Infectious Diseases, and; 9 Department of Medical Microbiology and Immunology, University of California, Davis, Sacramento, California, USA.; 10 Department of Microbiology, Immunology and Molecular Genetics, UCLA, Los Angeles, California, USA.

**Keywords:** Immunology, Infectious disease, Fungal infections, Immunotherapy, T cells

## Abstract

**BACKGROUND:**

Coccidioidomycosis ranges from self-limiting uncomplicated Valley fever (UVF) in most cases to life-threatening disseminated coccidioidomycosis (DCM) in rare individuals. A few patterns of immunologic deficits allowing for dissemination have been identified, although the specific defects in most individuals with DCM remain undefined. We hypothesized that chronic antigen exposure in DCM engenders a state of T cell exhaustion.

**METHODS:**

From a cohort of over 300 individuals with confirmed diagnoses of coccidioidomycosis, circulating T cell phenotypes were characterized via flow cytometry and *Coccidioides*-specific T cell responses were measured by activation-induced marker (AIM) assay.

**RESULTS:**

Male sex was significantly associated with disseminated disease (OR 2.5, 95% CI 1.5–4.0). A majority (52%) of individuals showed *Coccidioides*-specific T cell responses in our AIM assay. We noted a significant difference in patients sampled in the first year of diagnosis, where only 8% of patients with DCM had T cell responses during this time, as compared with 44% of individuals with UVF (*P* = 0.04). Among DCM patients with detectable AIM responses, CD4^+^ T cells demonstrated an exhausted phenotype with elevated PD-1 expression compared with UVF individuals. In vitro PD-1 blockade augmented IFN-γ production in most tested individuals with DCM.

**CONCLUSION:**

These findings suggest that dissemination may occur in some individuals during a period of impaired antigen-specific T cell activity. Importantly, these responses can be augmented in vitro by PD-1–blocking antibodies, supporting further study of immune checkpoint therapy as an adjunct to antifungal treatment in disseminated coccidioidomycosis.

**FUNDING:**

National Institute of Allergy and Infectious Diseases grants U19 AI166059 and R21 AI149654 and University of California Office of the President grant VFR-19-633386.

## Abstract 2

Researchers found that severe Valley fever is linked to a weakened immune response, pointing to treatments that could re-energize the body’s defenses.

## Introduction

Coccidioidomycosis comprises clinical presentations ranging from mild to catastrophic in patients who appear to be otherwise healthy, suggesting that yet unappreciated immunologic deficits may contribute to severe cases (1). *Coccidioides* is acquired by inhalation of environmental conidia, which then propagate in the host as spherules. More than half of individuals exposed to *Coccidioides* develop lifelong protective immunity without recognizable illness (2). Symptomatic disease most often presents as uncomplicated Valley fever (UVF): a self-limited pneumonia. Complicated pulmonary coccidioidomycosis (CPC), characterized by cavitation, respiratory failure, or persistent pulmonary disease despite treatment occurs less frequently and is associated with immunocompromising conditions such as diabetes and advanced age (3, 4). Extrapulmonary disseminated coccidioidomycosis (DCM) is the rarest and most severe presentation, occurring in only approximately 1% of cases and often involving lymphatic tissues, the musculoskeletal system, skin, or the meninges (5). Non-meningeal DCM is often chronic, requiring 3 or more years of antifungal therapy to achieve sustained remission, while meningitis is almost universally fatal without lifelong treatment (6, 7). Even with antifungal therapy, mortality for cases requiring ICU admission approaches 50% in contemporary cohorts (8). The burden of coccidioidomycosis is increasing, with recent incident rates estimated at 206,000–360,000/year (9). Immunomodulatory therapy is a promising approach to rescue antifungal refractory disease and hasten recovery in severe cases (10, 11).

Type 1 T helper (Th1) responses are central for control of *Coccidioides* infections, as seen through mouse models and vaccine studies (12). Th1 cells direct delayed-type hypersensitivity (DTH) reactions and augment the phagocytic killing of *Coccidioides* through the action of their key cytokine IFN-γ. Patients with coccidioidomycosis demonstrating erythema nodosum, a DTH-mediated panniculitis, have favorable outcomes (e.g., UVF) even among high-risk patient populations (13). On the other hand, impaired Th1 function leads to worse outcomes. Inborn errors of immunity affecting pathways critical for Th1 induction and effector function (such as IL-12 or IFN-γ signaling) are overrepresented in patients with DCM (10, 14, 15). Lymphocyte-suppressive medications for solid organ transplantation or rheumatologic disease are also associated with increased risk for DCM (14, 16). Patients with DCM are less likely to present with pulmonary nodules, suggesting an impaired granulomatous response at the time of infection (6).

Reliable detection of *Coccidioides*-specific T cells has proven challenging but previous studies suggest that the magnitude of Th1 responses is lower in severe disease presentations. In the early to mid-20th century, skin testing to measure DTH reactions against *Coccidioides* conidia or spherule-derived antigens was purportedly prognostic, with robust skin test responses predicting convalescence and anergic responses predicting disseminated disease and relapse (2, 17). More recent studies, however, have found no clear association between skin test results and disease severity or outcome, at least possibly due to heterogeneities in the skin test reagents (6, 18). In vitro studies, primarily employing the *C*. *posadasii* spherule–derived T27K antigen, have shown that patients with UVF exhibit greater T cell responses than those with DCM, as measured by proliferation (^3^H-thymidine uptake), IFN-γ production, and CD69 expression (19–22). The disparity in T cell responses between disease states may be most prominent during the first 6 to 12 months of infection, during which time patients with DCM interestingly mount exaggerated inflammatory responses with notable elevation of IL-10 and IL-6 (21, 23, 24). DCM is also associated with high-titer *Coccidioides*-specific antibodies that wane slowly with treatment (25). Thus, while patients with DCM mount robust immunity, the response appears biased toward exaggerated humoral inflammation but impaired T cell activity.

T cell “exhaustion” arises in chronic disease states where there is prolonged exposure to antigen, such as in chronic infections or cancers. Chronic T cell receptor signaling imposes epigenetic changes that impair cytokine production and cytotoxic killing (26, 27). Monoclonal antibodies that block the inhibitory receptor PD-1 can transiently restore the effector functions of exhausted T cell responses, and have become the standard of care for several cancers (28). Development of granulomata in fungal infections arise from local Type 2 immune responses (29) and allow fungi to persist in a latent form for years or decades (30). The impaired Th1 responses of early DCM may promote T cell exhaustion by establishing a long-lasting antigenic depot within the immunosuppressive granulomatous milieu. Mouse models demonstrate that *Coccidioides* induces myeloid PD-L1 expression, suggesting that targeting the PD-1/PD-L1 pathway may have therapeutic merit (31). PD-1 blockade has shown promise in mouse models of dimorphic fungal infection and several human cases of invasive mold infection (32–38).

We hypothesized that the chronic disease state of DCM, characterized by prolonged exposure to *Coccidioides* antigens (as potentially evidenced by prolonged elevation of antibody titers) and dysfunctional T cell responses may create an environment that fosters T cell exhaustion. In this study, we characterized peripheral T cell phenotypes in a large cohort of individuals with a range of *Coccidioides* disease and measured *Coccidioides*-specific T cell responses by antigen-induced marker (AIM) assay. In DCM, T cell responses correlated with later stages of disease and increased expression of exhaustion markers. Our findings suggest that in some patients with DCM, *Coccidioides*-specific T cells are exhausted and can be augmented by PD-1 blockade.

## Results

### Cohort characteristics.

Three hundred and ten individuals with confirmed diagnosis of coccidioidomycosis were recruited. Clinical disease category and demographics are reported in Table 1. Age, sex, self-identified race and ethnicity (SIRE), and date of initial *Coccidioides* diagnosis were provided by individuals at recruitment. Coccidioidomycosis severity for each individual was categorized using the 5-level numeric classification described by Krogstad and colleagues (1, asymptomatic; 2, uncomplicated pulmonary; 3, complicated pulmonary; 4, non-meningeal disseminated; 5, meningitis) (1). Complicated pulmonary coccidioidomycosis (CPC), non-meningeal disseminated coccidioidomycosis (NMDCM), and *Coccidioides* meningitis (CM) were further stratified into subcategories. CPC was heterogeneous, comprising approximately equal proportions of simple cavitary disease (3A, 41%) and persistent symptoms despite 6 months of therapy (3C, 46%) with few cases of respiratory failure (3D, 10%) or fibrocavitary disease (3B, 3%). For NMDCM, most had dissemination to extrapulmonary organs or deep tissue sites (4B, 88%), while only a few showed dissemination to skin only (4A, 12%). Most individuals with CM had isolated meningeal disease (5A, 85%), while fewer had additional dissemination to skin (5B, 4%) or deeper tissue sites (5C, 11%).

We investigated whether demographic variables were associated with severe disease states by consolidating individuals into 2 broader groups: UVF (categories 1 and 2) and DCM (categories 4 and 5). CPC cases were excluded from these analyses due to the clinical heterogeneity of this group, which suffered more complicated syndromes than the self-limited UVF, yet without extrapulmonary dissemination. Male sex was significantly associated with DCM (Fisher’s exact test, *P* = 0.007; OR 2.5, 95% CI 1.5–4.0, *P* < 0.001; Supplemental Figure 1; supplemental material available online with this article; https://doi.org/10.1172/jci.insight.203270DS1) as other studies also observed (2). Previous studies suggest that persons identifying as Hispanic, Black, or Asian have increased risk for DCM relative to those identifying as White (39, 40). However, our cohort showed that SIRE of Hispanic or Latino was associated with a decreased risk for DCM (OR 0.4, 95% CI 0.2–0.7) while risk was increased among non-Hispanic White (OR 3.1, 95% CI 1.5–6.2) and Black (OR 4.2, 95% CI 1.2–22) but not Asian individuals (Supplemental Figure 1). Regarding age, DCM individuals were older than UVF at recruitment (median 50 years, IQR 34–58 vs. 43, IQR 33–56, *P* = 0.05 by Mann-Whitney *U* test) but age at diagnosis was not different (median 41 years, IQR 28–50 vs. 40 years, IQR 20–53, *P* = 0.6 by Mann-Whitney *U* test). Notably, individuals with UVF were recruited significantly more proximally to their dates of diagnosis than those with DCM (median 9 months, IQR 3–46 vs. 57 months, IQR 20–129, *P* < 0.0001 by Mann-Whitney *U* test). Taken together, these data show our cohort comprises a typical and diverse spectrum of demographics and clinical phenotypes.

### T cell phenotype in DCM suggests exhausted immune state.

**Figure 1:**
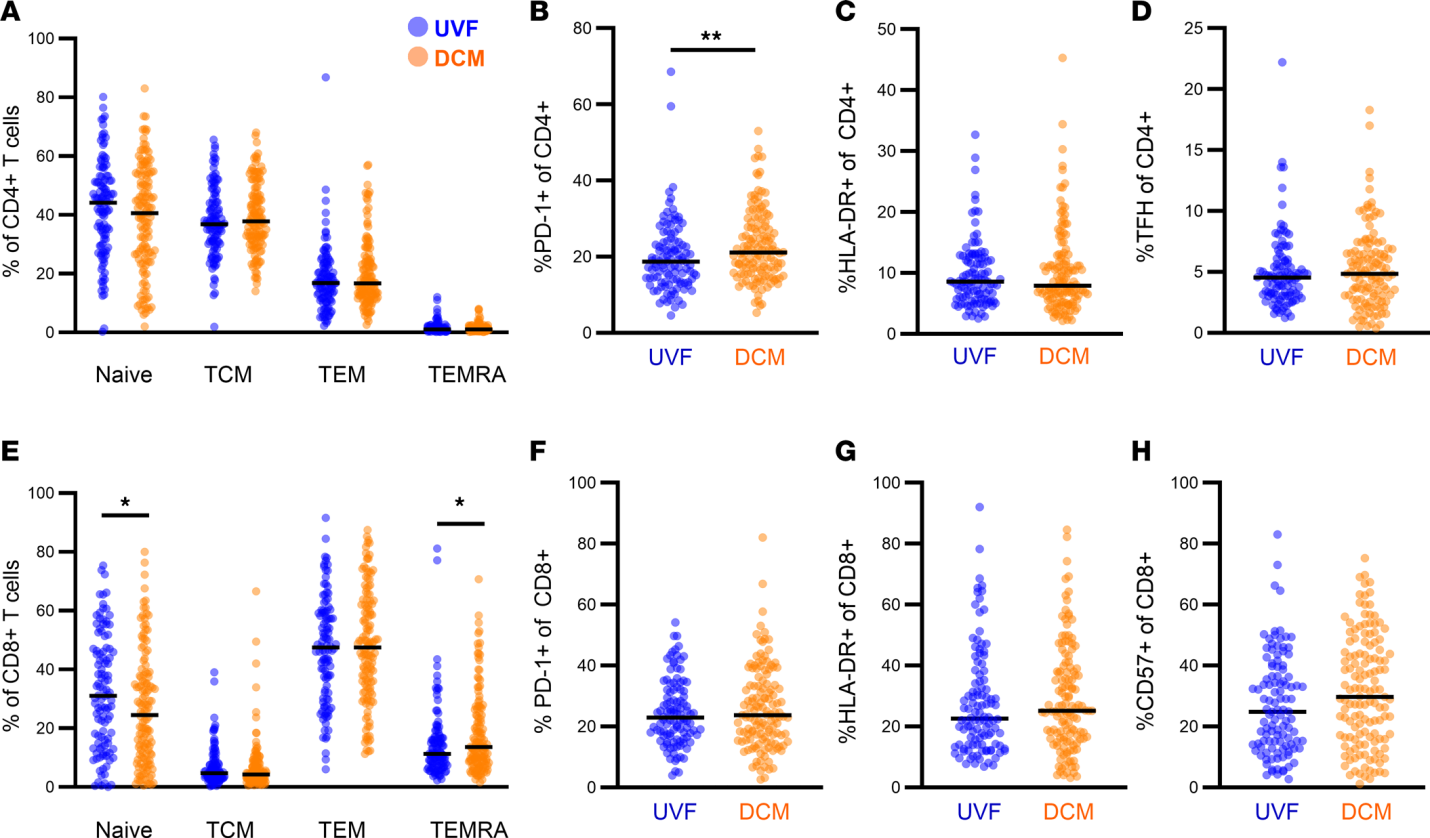
PBMC T cell phenotype of UVF and DCM. PBMCs from patients with UVF (blue, *N* = 108) and DCM (orange, *N* = 130) were phenotyped by flow cytometry. (**A** and **E**) Naive (CD45RA^+^CCR7^+^), central memory (TCM, CD45RA^–^CCR7^+^), effector memory (TEM, CD45RA^–^CCR7^–^), and TEMRA (CD45RA^+^CCR7^–^) subsets of (**A**) CD4^+^ and (**E**) CD8^+^ T cells. (**B**–**D**) Percentage of (**B**) PD-1^+^, (**C**) HLA-DR^+^, and (**E**) T follicular helper (TFH, PD-1^+^CXCR5^+^) CD4^+^ T cells. (**F–H**) percentage of (**F**) PD-1^+^, (**G**) HLA-DR^+^, and (**H**) CD57^+^ CD8^+^ T cells. Bars indicate the mean. Statistical comparisons by Mann-Whitney *U* test, unadjusted. *P < 0.05, **P < 0.01.

We assessed whether biomarkers of peripheral T cell differentiation and activation differed between disease severities. Naive (CD45RA^+^CCR7^+^) T cells are quiescent and upon TCR engagement proliferate and differentiate into effector T cells. Circulating central memory T (TCM, CD45RO^+^CCR7^+^) cells provide a long-lived reservoir with high proliferative potential upon antigen reexposure and circulate between lymphoid tissues to endow follicular and extrafollicular help. Effector memory T (TEM, CD45RO^+^CCR7^–^) cells traffic between the circulation and peripheral tissues and provide fast cytokine recall responses. The role of CD4^+^ terminally differentiated effector memory RA (TEMRA) cells is less clear in fungal infections, but they likely represent effector memory cells in a more terminally differentiated state. By flow cytometry we measured T cell memory subsets (naive, TCM, TEM, TEMRA) and activation/exhaustion markers HLA-DR, PD-1, and CD57 in peripheral blood CD4^+^ and CD8^+^ T cells (Figure 1 and Supplemental Figure 2). We found that CD4^+^ T cell memory subsets were not significantly different between individuals with DCM or UVF. However, the DCM group showed a trend toward decreased CD4^+^ naive T cells compared with UVF (median 41%, IQR 26–54 vs. 44%, IQR 30–53, *P* = 0.2 by Mann-Whitney *U* test, Figure 1A). CD8^+^ T cell populations trended similarly to CD4^+^ T cells. Naive CD8^+^ T cells were fewer in DCM compared with UVF (median 24%, IQR 11–38 vs. 31%, IQR 15–46, *P* = 0.03) and TEMRA populations were larger (median 9%, IQR 2–14 vs. 7%, IQR 2–11, *P* = 0.04) (Figure 1E). These results show that DCM does not entail a quantitative immunodeficiency among helper T cells.

PD-1 expression on CD4^+^ T cells was significantly greater in the DCM group relative to UVF (median 21%, IQR 16–28 vs. 19%, IQR 13–24, *P* = 0.007; Figure 1B). PD-1 on T cells can demonstrate the exhausted phenotype in states of chronic infection weeks or more after acute infection (41, 42), but PD-1 can also appear as a short-term activation marker on T cells for a few hours to days after TCR stimulation. We evaluated whether expression of PD-1 could have indicated recent stimulation by measuring the activation marker HLA-DR on CD4+ T cells (43, 44), which was not significantly different between the groups (median 8%, IQR 6–13 in DCM vs. 9%, IQR 6–12 in UVF; Figure 1C). PD-1 is also expressed on circulating follicular helper (TFH) CD4^+^ T cells and facilitates productive interactions between T and B cells (45). *Coccidioides*-specific antibody titers are often dramatically elevated in patients with DCM. To assess whether the increased PD-1 expression in this group was driven by an expanded TFH subset, we measured CD4^+^PD-1^+^CXCR5^+^ circulating TFH cells, which were not significantly different between DCM and UVF (median 5, IQR 3–7 vs. 5, IQR 3–6; Figure 1D). These results show that the population of PD-1–expressing CD4^+^ T cells is not attributable to TFH or recent activation, and thus PD-1 expression may represent exhaustion.

For CD8+ T cells, PD-1 expression was no different between DCM and UVF (median 24%, IQR 16–34 vs. 23%, IQR 17–32, P = 0.7; Figure 1G), but the senescence marker CD57 trended slightly higher in DCM (median 30%, IQR 16–46 vs. 25%, IQR 14–39, P = 0.05; Figure 1H).

T cell phenotypes thus suggested a more differentiated (i.e., less naive) and exhausted state in DCM relative to UVF, with increased PD-1 expression among CD4^+^ T cells and decreased naive T cells. Since individuals with DCM and UVF also varied by age, sex, and time since diagnosis, we assessed whether these variables confounded our findings or identified interesting subgroups. PD-1 expression on CD4^+^ T cells showed a positive but statistically insignificant correlation with age in both UVF and DCM, with a stronger correlation among DCM relative to UVF (Spearman’s *r* = 0.098 vs. 0.026) (Supplemental Figure 3, A and B). We did not identify significant correlations between PD-1 expression on CD4^+^ T cells with time since diagnosis or sex (Supplemental Figure 3, C–F). As some of the variance in PD-1 expression could be attributed to the age of the individual, we assessed the variance of all measured phenotypic markers with age by linear regression. Across the entire cohort, age associated similarly with CD4^+^PD-1^+^ cells (*r*^2^ = 0.014) as compared with CD4^+^HLA-DR^+^ cells (*r*^2^ = 0.023), while other subsets such as CD4^+^ TCM (*r*^2^ = 0.11), CD8^+^ naive (*r*^2^ = 0.24), CD8^+^HLA-DR^+^ (*r*^2^ = 0.074), and CD8^+^CD57^+^ (*r*^2^ = 0.061) showed a much stronger association with age (Supplemental Figure 4). These results show that age does not sufficiently explain the variance in PD-1 expression on CD4^+^ T cells between individuals with DCM and UVF. In cancer, certain markers found on PBMCs prior to treatment with an anti–PD-1 antibody were associated with favorable responses, including ones we saw in individuals with DCM: increased PD1 expression, increased CD57 expression on CD8^+^ T cells, and increased CD4^+^ memory populations relative to naive (47, 48). Taken together, our findings of phenotypic differences in T cells suggested an exhausted state.

### AIM assay for measurement of Coccidioides-specific T cell responses.

**Figure 2:**
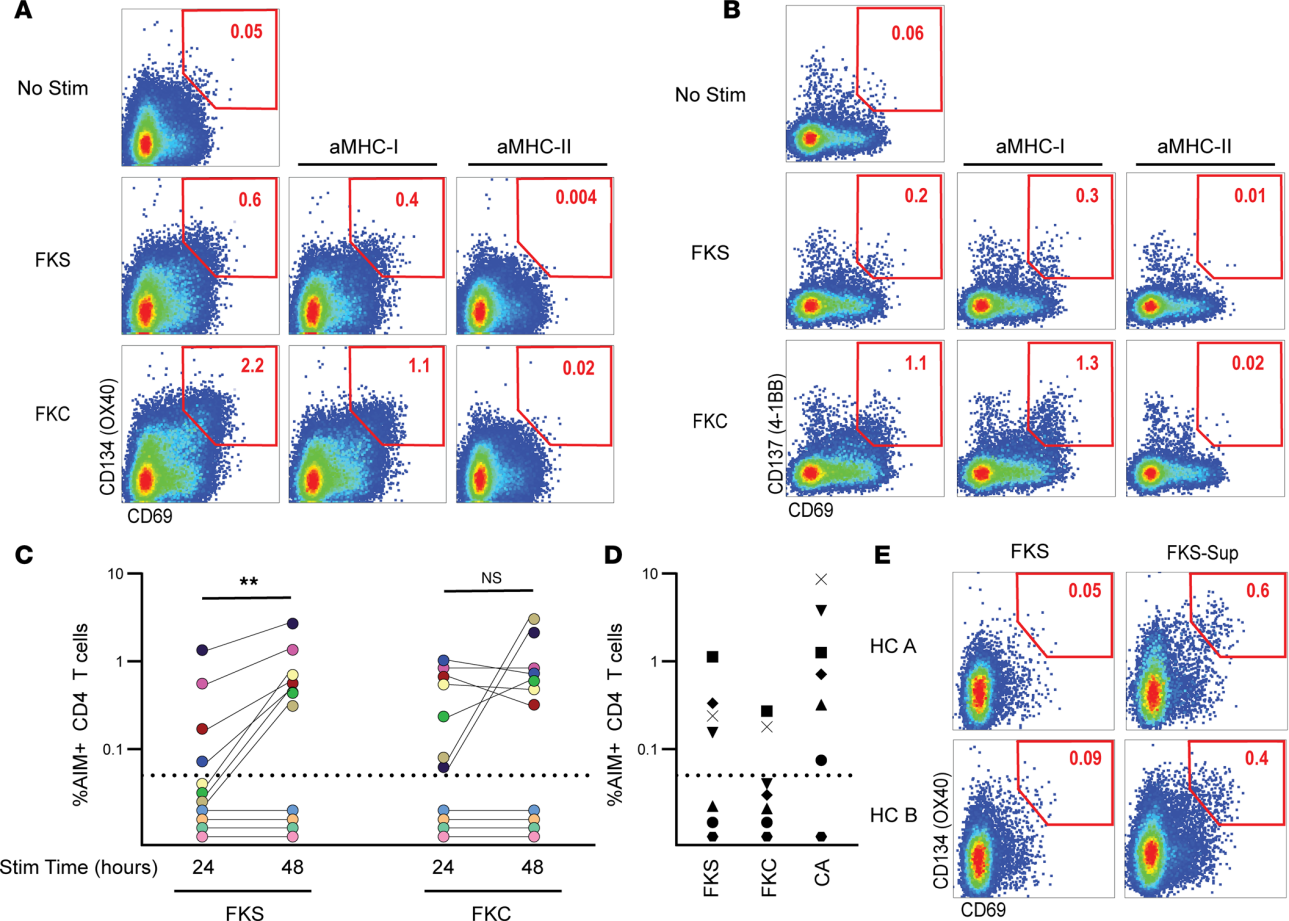
AIM assay to identify *Coccidioides*-specific T cell responses. (**A** and **B**) PBMCs were incubated with *Coccidioides* formalin-killed spherules (FKS), formalin-killed conidia (FKC), or media alone (No Stim) with or without MHC-I–blocking antibody (αMHC-I) or MHC-II–blocking antibody (αMHC-II) for 24 hours. AIM-positive populations are gated for (**A**) CD4^+^ T cells (CD69^+^OX40^+^) and (**B**) CD8^+^ T cells (CD69^+^CD137^+^). (**C**) PBMCs from 11 individuals with coccidioidomycosis (mixed UVF and DCM) were simulated with FKS and FKC for 24 and 48 hours. Percentage AIM-positive CD4^+^ T cells with unstimulated background subtracted are presented. Colors denote unique individuals. Comparisons by Wilcoxon’s matched pairs rank-sum test. **P < 0.01. (**D**) PBMCs from 7 healthy control donors (HC) stimulated with FKC, FKS, or candida antigen (CA) for 24 hours. Symbols denote unique individuals. For **C** and **D**, values below dotted line (0.05%) are negative. (**E**) PBMCs from 2 HC (HC A and HC B) were stimulated for 48 hours with FKS alone or supernatant collected from cultures of *Coccidioides*-infected individuals’ PBMCs stimulated with FKS for 48 hours (FKS-sup). The AIM-positive population of CD4^+^ T cells is gated.

We next sought to compare functional, *Coccidioides*-specific T cell responses between UVF and DCM. *Coccidioides*-specific T cell responses were measured by AIM assay (49). PBMCs were stimulated separately with formalin-killed spherules (FKS) or formalin-killed conidia (FKC) derived from the *Coccidioides* *immitis* RS strain. Activated CD4^+^ T cells were identified by coexpression of CD69 and CD134 (OX40), and activated CD8^+^ T cells by both CD69 and CD137 (4-1BB) (Supplemental Figure 5). AIM assays allow for the identification of diverse populations of antigen-specific CD4^+^ and CD8^+^ T cells specific for linear peptide antigens (e.g., SARS-CoV-2 and HCV) and more complex antigen preparations such as lysates of *Mycobacterium* *tuberculosis* and *Trypanosoma* *cruzi* (50–53). T cells are reported to express AIM markers both in response to TCR engagement with cognate peptide-MHC complexes and, when cultured over longer periods, antigen-independent “bystander” activation by stimulatory cytokines (54). We tested whether AIM responses to FKC and FKS were TCR dependent by stimulating PBMCs in the presence of MHC-I– and MHC-II–blocking antibodies (Figure 2, A and B). Both CD4^+^ and CD8^+^ T cell responses were fully suppressed by MHC-II blockade but not MHC-I blockade, suggesting that FKS- and FKC-responsive CD4^+^ T cells were antigen-specific, while bystander activation drove CD8^+^ T cell responses. This finding is consistent with extracellular antigen processing predominantly utilizing endosomal pathways and presentation on MHC-II molecules. Because of this finding and because the role of helper T cells is much better defined in fungal immunity, we therefore directed our focus to CD4^+^ T cells.

To assess the optimal stimulation period for these antigens, we compared T cell responses against FKS and FKC over 24-hour and 48-hour incubation periods (Figure 2C). FKS-specific responses were greater at 48 hours of stimulation than 24 hours (*P* = 0.008, Wilcoxon’s matched pairs rank-sum test), while FKC responses were not significantly different between the 2 time points. Seven of the 11 patients (64%) demonstrated a detectable response to any antigen across both time points, and all of these showed positive FKC-specific responses at 24 hours. These results show that 24-hour stimulation with FKC was adequate to detect cocci-specific T cell responses in over half the patients, and FKS required a longer stimulation period.

To assess the specificity of the responses to FKS and FKC, we examined the AIM assay in PBMCs from endemic-region healthy control donors (HC, *N* = 7) with an unknown history of *Coccidioides* exposure (Figure 2D). Four HC (57%) showed a positive response to FKS, while 2 (29%) responded to FKC. Interestingly, the 4 HC with FKS-specific responses also showed high T cell responses to stimulation with candida antigen. In vitro T cell cross-reactivity between *Coccidioides* with other endemic mycoses, including *Histoplasma* and *Blastomyces*, has been described, but whether *Coccidioides*-specific T cells cross-react with Candida is not known (55). We then attempted to determine whether the increased FKS-specific responses at 48 hours were driven by delayed T cell activation or bystander activation. PBMCs from 2 HC were stimulated with FKS alone for 48 hours or with supernatant (FKS-Sup) collected from AIM-positive individuals stimulated with FKS for 48 hours. FKS-Sup–stimulated HC PBMCs showed a greater percentage of AIM^+^CD4^+^ T cells than FKS alone (Figure 2E), suggesting that soluble factors produced by FKS-stimulated cells could augment T cell responses over extended incubation periods. Therefore, while the extended 48-hour stimulation period was necessary to reliably detect FKS-specific responses, some of these responses could also accumulate the effects of bystander T cell activation.

The finding of bystander effect and possible cross-reactivity with other fungal antigens does not diminish the relevance of spherule-specific T cell responses. *Coccidioides* is transmitted as conidia but propagates within infected hosts as spherules, and these 2 forms demonstrate unique expression patterns of their transcriptomes and proteomes (56). It is long established that different phases of the *Coccidioides* life cycle elicit different T cell responses (57); however, the exact antigens that are unique or overlapping across phases and their contributions to virulence have not been fully elaborated (58, 59). We therefore concluded that the presence or absence of *Coccidioides*-specific T cell responses could be adequately assessed by either FKC at 24 or 48 hours of stimulation, or FKS at 48 hours stimulation, though the absolute magnitude of response may differ by the method employed.

### T cell responses are impaired early in disseminated Coccidioides.

**Figure 3:**
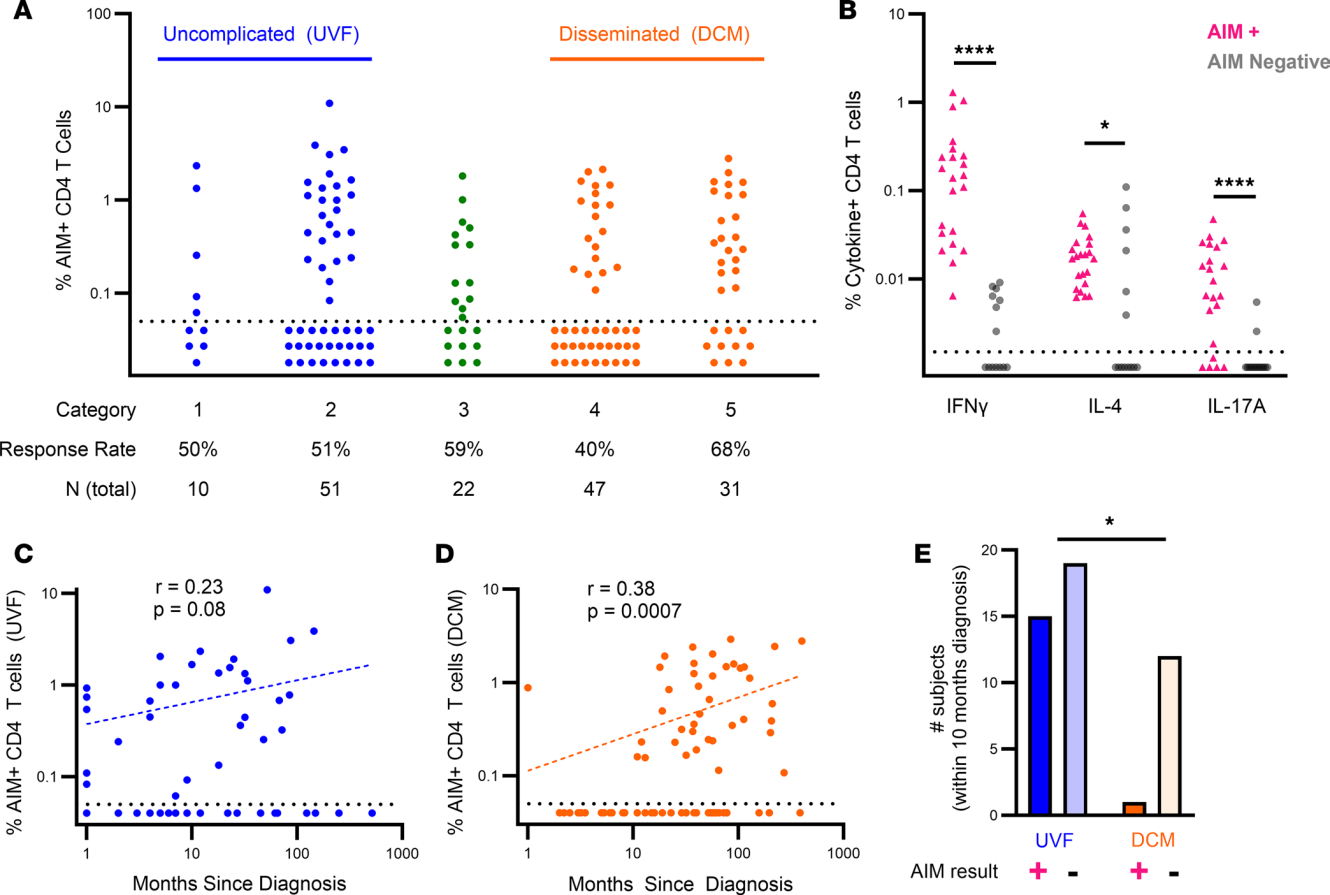
*Coccidioides*-specific T cell responses for 161 individuals by disease category. (**A**) AIM responses stratified by *Coccidioides* disease category. Limit of detection = 0.05%, values below dotted line are negative. (**B**) IFN-γ, IL-4, and IL-17 production of CD4^+^ T cells stimulated with FKS or FKC from individuals with positive *Coccidioides*-specific T cell responses by AIM assay (AIM^+^, pink, *N* = 22) or no response (AIM negative, grey, *N* = 14). Comparisons by Wilcoxon signed rank test. (**C** and **D**), Spearman correlation of *Coccidioides*-specific T cell responses by AIM and time since diagnosis for (**C**) UVF and (**D**) DCM. (**E**) Number of individuals with UVF or DCM within 10 months of diagnosis with positive or negative *Coccidioides*-specific T cell responses by AIM assay. Fisher’s exact test. *P < 0.05; ****P < 0.0005.

We surveyed *Coccidioides*-specific T cell responses by AIM assay in 161 individuals from various disease categories across our cohort (Figure 3A). Positive responses were detected in 52% of individuals (*N* = 84 of 161). By disease category, the response rates were 50% for asymptomatic disease (ASX, 5 of 10), 51% for uncomplicated pulmonary (UPC, 26 of 51), 59% for CPC (13 of 22), 40% for NMDCM (19 of 47), and 68% for CM (21 of 31). Given the low response rate, we sought to validate that the AIM assay correlated with functional T cell responses. We tested 22 AIM-positive and 14 AIM-negative individuals for production of IFN-γ, IL-4, and IL-17A in response to stimulation with *Coccidioides* antigens (Figure 3B and Supplemental Figure 6). FKS and FKC provoked similar responses, and cytokine production was almost universally absent in individuals who lacked an AIM response. This association between AIM and cytokine readouts for detection of *Coccidioides*-specific T cell responses did not vary by disease category.

Most of our individuals with DCM were recruited years after their initial diagnosis and those with UVF were mostly recruited within 1 year. To investigate whether the proximity of blood sampling to diagnosis affected T cell responses, we compared AIM responses by time since *Coccidioides* diagnosis. The result revealed a strikingly low response rate among individuals with DCM recruited early in disease (Figure 3, C and D); only 1 of 13 (8%) individuals with DCM within 10 months of diagnosis demonstrated a positive AIM response. In contrast, positive responses were detected for 15 of 34 (44%) individuals with UVF within 10 months of diagnosis (*P* = 0.04, Fisher’s exact test; Figure 3E). These results reveal an almost 1-year refractory period of *Coccidioides* antigen recognition by CD4^+^ T cells for individuals with DCM but not UVF.

We compared AIM responses by age and sex, as these variables also differed between UVF and DCM (Supplemental Figure 7). AIM responses did not vary significantly with age (Supplemental Figure 7, A and B). For both UVF and DCM, a larger percentage of females were AIM responders than males (Supplemental Figure 7, C and D). This sex-dependent difference in response rate was more was pronounced in DCM (female, 68%; male, 45%) than UVF (female, 55%; male, 47%) but was not statistically significant (*P* = 0.09, 0.6, respectively, by Fisher’s exact test).

### Patients with DCM with detectable T cell responses show an exhausted phenotype and respond to checkpoint blockade in vitro.

**Figure 4:**
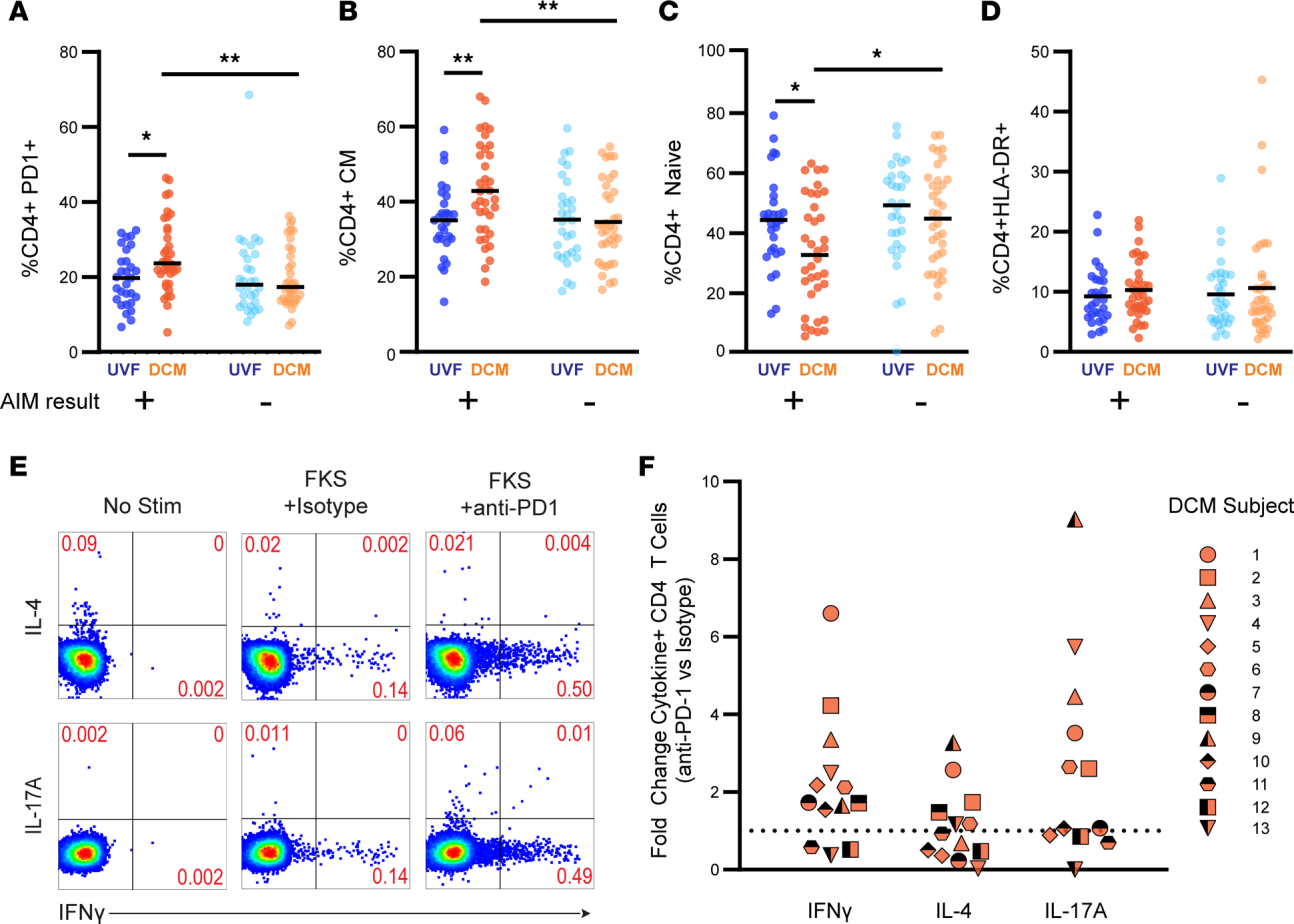
Checkpoint blockade augments *Coccidioides*-specific T cell responses in some individuals with DCM. (**A**–**D**) PBMC CD4^+^ T cell phenotypes were compared between AIM-positive UVF (dark blue, *N* = 29), AIM-positive DCM (dark orange, *N* = 34), AIM-negative UVF (light blue *N* = 30), and AIM-negative DCM (light orange *N* = 39) individuals. We specifically assessed (**A**) CD4^+^PD-1^+^, (**B**) CD4^+^ naive, (**C**) CD4^+^ central memory (CM), and (**D**) CD4^+^HLA-DR^+^ populations. Statistical comparisons by Mann-Whitney *U* test. *P < 0.05; **P < 0.01. (**E** and **F**) PBMCs from *N* = 13 AIM-positive DCM individuals were cultured with media alone (No Stim), FKS with isotype control antibody, or FKS with anti–PD-1 antibody. IFN-γ, IL-4, and IL-17A production was measured by intracellular staining and flow cytometry. (**E**) Representative patient data. (**F**) Fold change in cytokine production; each symbol represents 1 patient.

Having identified an apparent deficit in the induction of T cell responses in DCM, we then assessed whether patients with DCM with T cell responses (i.e., AIM-positive) demonstrated functional T cell exhaustion. Revisiting the PBMC T cell phenotypes presented in Figure 1 among just the AIM-positive individuals, more CD4^+^ T cells expressed PD-1 in AIM-positive DCM individuals compared with AIM-positive UVF individuals (median = 24% vs. 20%, *P* = 0.01 by Mann-Whitney *U* test; Figure 4A). Interestingly, CD4^+^ T cell PD-1 expression was significantly lower in the AIM-negative DCM group (median = 17%, *P* = 0.005 by Mann-Whitney *U* test; Figure 4A), suggesting that the increased PD-1 expression in DCM may be driven by *Coccidioides*-specific immunity. Phenotypes also showed decreased naive CD4^+^ T cells and increased CD4^+^ TCM cells in AIM-positive DCM compared with AIM-negative DCM and UVF, consistent with a more antigen-experienced state, without significantly different HLA-DR expression (Figure 4, B–D).

We tested whether *Coccidioides*-specific T cell effector responses could be augmented by checkpoint blockade. PBMCs from 13 AIM-positive DCM individuals were stimulated with FKS or FKC in the presence of PD-1 inhibitor or isotype control (Figure 4, E and F). Ten individuals (77%) showed an increase in CD4^+^ T cell IFN-γ production with PD-1 blockade, and in 6 individuals (46%) the increase was 2-fold or greater (Figure 4F). Increases in IL-17A production, and to a lesser extent IL-4 production, were observed in some individuals as well. Checkpoint blockade did not boost cytokine production in AIM-negative individuals.

We attempted to identify phenotypic or demographic correlates associated with response to checkpoint blockade. Fold change in IFN-γ production against *Coccidioides* antigen with PD-1 blockade was compared to phenotypic markers, AIM response, age, and time since infection by linear regression (Supplemental Figure 8). The 2 highest responders (representing IFN-γ fold changes of 4.2 and 6.6) were both female with CD4^+^ T cell PD-1 expression of greater than 40%; the 3 nonresponders were all male. However, no phenotypic or demographic variable assessed showed significant variation with response to PD-1 blockade.

## Discussion

We investigated peripheral T cell phenotypes and *Coccidioides*-specific CD4^+^ T cell responses in a large cohort with diverse manifestations of coccidioidomycosis. T cells in DCM trended toward an exhausted, antigen-experienced phenotype with expanded memory populations and increased expression of PD-1 on CD4^+^ T cells that was not attributable to age. T cell responses against a set of *Coccidioides* antigenic preparations measured by AIM assay showed a nearly 1-year refractory period in DCM. PD-1 expression was highest among DCM individuals with detectable T cell responses, suggesting an association between exhausted phenotypes and *Coccidioides-*specific immunity. PD-1 blockade boosted T cell cytokine production against *Coccidioides* antigens in most DCM individuals. Our findings support the notion that exhaustion contributes to T cell dysfunction in some cases of DCM and warrants further exploration of adjunct checkpoint inhibitor therapy for severe cases.

In our cohort, male sex was associated with increased risk for disseminated disease, as was non-Hispanic White or Black (SIRE). Male sex is an established risk factor for coccidioidomycosis as well as a broad host of viral, bacterial, and fungal infections. While the increased susceptibility of men to coccidioidomycosis is not fully understood, testosterone has been described to suppress Th1 differentiation and function, which may partially explain sex differences in disease risk (60). African and Filipino ancestry have been consistently associated with more severe *Coccidioides* disease states in previous studies (61). Our cohort recapitulates increased risk of DCM among individuals identifying as Black (SIRE), despite representing a minority of individuals (*N* = 18). We did not specifically elicit Filipino ancestry from individuals in this study. The findings of decreased risk for DCM among Hispanic or Latino individuals and increased risk among non-Hispanic White individuals is contrary to previous findings but may reflect that our cohort is mostly Hispanic or Latino (*N* = 198 of 310, 64%).

We expected that all immunocompetent patients would mount a detectable T cell response to at least some *Coccidioides* antigens. However, the history of assessing cellular immunity in coccidioidomycosis is fraught with 2 major concerns: inadequate sources of *Coccidioides* antigens and inadequate readouts. For this reason, we introduced the AIM assay here using antigenic preparations derived from *C*. *immitis* RS strain spherules and conidia.

Other sources of *Coccidioides* antigen have been employed over the years with modest-to-poor elicitation of T cell responses. The *C*. *posadasii* spherule lysate T27K has been used in assays measuring the lone activation marker CD69 and cytokine production in endemic healthy controls, nonendemic healthy controls, and *Coccidioides* patients with variable severity of disease (19, 21, 62, 63). Although response rates in these studies are not reported, in vitro stimulation with T27K showed 83%–85% agreement with coccidioidin skin testing (64). More remote studies in the 1970s also showed that in vitro lymphocyte stimulation with mycelial or spherule derived lysates largely agree with skin test results (22). Skin testing (namely, delayed-type hypersensitivity [DTH]) with spherule-derived antigen was originally described to demonstrate greater than 98% sensitivity and specificity for patients with pulmonary coccidioidomycosis, suggesting that in vitro responses to *Coccidioides* antigens should be much higher than the 52% than we observed in our cohort (65). However, more recent studies suggest that in practice, only 55%–72% of patients with a *Coccidioides* diagnosis have a positive DTH (18, 66). Peptide antigens are also under development for diagnostic testing. Kala and colleagues recently designed a mix of 108 peptides derived from *Coccidioides* proteins that induce T cell responses in mice and are upregulated during the parasitic growth phase (67). However, even this more modern approach did not detect a response in all individuals with coccidioidomycosis.

Antifungal therapy reduces fungal burden and is postulated to decrease skin test sensitivity, suggesting that prolonged untreated infection may be necessary to develop detectable T cell responses in some cases (18). T cells may also be sequestered to lymph nodes or infected tissue sites during acute disease. For example, in CM, *Coccidioides*-specific T cells can durably be detected in cerebrospinal fluid while absent from peripheral blood (68). In our hands, the AIM assay in CD4^+^ T cells passed important quality measures, including dependency on MHC-II interactions and correlation with cytokine production. We noted a difference between antigenic preparations, with FKC eliciting responses in 24 hours, while FKS required 48 hours for consistent results. This difference may be due to differential accessibility of antigens and the larger size of spherules, which may require longer incubation for effective antigen processing and presentation (69). Whether the difficulty in reliably detecting these responses in peripheral blood is due to tissue compartmentalization, anergy, lack of optimized antigen, or inadequate assays warrants further study.

*Coccidioides*-specific T cell responses within our cohort showed a profoundly low response rate among DCM individuals sampled within 10 months of diagnosis. This delay in developing productive T cell responses offers a window for the infection to disseminate, as dissemination most often occurs within 6 months of diagnosis (6). We know from many animal models and vaccine studies that an adequate T cell response during the early phase of disease is critical to prevent dissemination. In cancer, T cell exhaustion begins to show a relative shift from effector to exhaustion pools as early as 6 hours after exposure (70), although it is well appreciated that a few weeks of chronic antigen exposure are needed for the full exhaustion phenotype. In this study we suggest a relationship between T cell exhaustion and hypo-responsiveness in DCM. Weak T cell responses have been reported over many decades, including the identification of weaker T27K-specific T cell responses in DCM compared with UVF during the first 5 and 12 months of infection by CD69 expression and whole-blood IFN-γ production, respectively (21, 23). Unfortunately, the low response rate to *Coccidioides* antigens detected by our assay, while not unexpected in the context of previous studies, is a major caveat that limits decisive interpretation of these results.

Our observation of exhausted T cell signatures in some individuals with DCM invokes CD4^+^ T cell exhaustion, which is less well understood than CD8^+^ T cell exhaustion. Initial descriptions of the molecular signatures underlying T cell exhaustion were borne from the study of CD8^+^ T cells in CD4^+^ T cell–depleted mouse models of chronic viral infection (71). Studies of CD4^+^ T cells in this same model showed some overlapping signatures with CD8^+^ T cell exhaustion, but with greater heterogeneity and distinct expression patterns. Relative to CD8^+^ T cells, exhausted CD4^+^ T cells show more sustained high levels of PD-1 expression during chronic infection, similar to the exhausted DCM phenotype in our cohort (72). CD8^+^ T cell exhaustion is emphasized in cancer due to the lack of MHC-II expression on most tumors and the aim of augmenting cytotoxic responses in this setting. However, CD4^+^ T cells are relevant for checkpoint inhibitor therapy as well (73). Exhausted tumor-specific CD4^+^ T cells can be found in solid malignancies and show increased functional responses with checkpoint inhibitor therapy (74). The role of CD4^+^ T cell exhaustion is more clearly defined in MHC-II–expressing tumors such as Hodgkin lymphoma, wherein tumor infiltrating lymphocyte populations are rich in CD4^+^ T cells expressing classical markers of exhaustion (i.e., PD-1 and TOX) and show enhanced antitumor activity with PD-1 blockade (75, 76). Interpretation of PD-1 expression on CD4^+^ T cells is also muddied by the role of PD-1 in regulating TFH interactions with B cells and the activity of CD4^+^ regulatory T cells (Tregs), which function in parallel with exhaustion to dampen potentially harmful inflammatory responses. Indeed, molecular analyses reveal that TFH and Treg gene expression patterns partially overlap with that of exhausted CD4^+^ T cells (72, 77). Our findings here only present an association between PD-1 expression, antigen-experienced T cell populations, and the chronic disease state of DCM that presents an opportune milieu to induce exhaustion. More extensive molecular interrogation of *Coccidioides*-specific CD4^+^ T cells in uncomplicated and disseminated disease is necessary to define exhaustion in this setting.

Our 2 key findings, T cell refractoriness in early DCM and T cell exhaustion in later disease, may represent either related or independent phenomena. T cell exhaustion is attributed to chronic antigen stimulation in a dysregulated microenvironment. Impaired antigen-presenting cell function, immune evasion by the fungus, or fungal toxicity to immune cells (78, 79) could cause dissemination, and resulting failure to clear the burgeoning antigenic load could drive exhaustion. This model of exhaustion as a consequence of dissemination is concordant with our results. Alternatively, exhaustion in early disease may permit dissemination. Supporting this hypothesis is evidence that exhausted T cells are detectable as early a few hours to days after infection (70, 80) and murine models of histoplasma or aspergillus show improved survival when anti–PD-1 antibodies are administered within 1 day of infection (81, 82). Further studies of the clonal dynamics of T cells may help clarify whether exhaustion is a cause or consequence of DCM.

Exhaustion is likely a protective adaptation in chronic infection that prevents excessive immunopathology (83). Some immunocompetent patients with cryptococcal meningitis experience a paradoxical exacerbation of symptoms after antifungal therapy due to overactive T cell responses, similar to the immune reconstitution inflammatory syndrome (IRIS) seen in people with HIV (84, 85). IRIS has not been recognized as a feature of coccidioidomycosis in immunocompetent patients, however inflammatory reactions such as hemophagocytic lymphohistiocytosis have been rarely observed (86). Corticosteroids are occasionally used for suspected *Coccidioides*-related vasculitis and may reduce the risk of repeat infarction, but this practice is controversial and corticosteroid use in CM is associated with substantially increased mortality (87–89). Furthermore, vasculitic presentations often occur with elevated biomarkers of disease activity and histologic infiltration of vascular walls by *Coccidioides* pathogens (90, 91). PD-1 blockade frequently causes immune-related adverse events that manifest as autoimmune organ injury, but is not associated with IRIS-like systemic inflammatory reactions (92). We propose that in the modern age with effective antifungals to reduce pathogen burden, exhaustion-mediated attenuation of the *Coccidioides*-specific T cells necessary to contain and eradicate disease is maladaptive.

Most patients with DCM do not have a clearly identified immune defect to explain their failure to control disease. We showed here that many of these individuals have deficient T cell responses that may be augmented by adjunct immunomodulation. Immunotherapy for *Coccidioides* was first described in the 1970s with the use of Transfer Factor, which reportedly led to clinical improvement and augmentation of lymphocyte responses (93, 94). IFN-γ then emerged in the 21st century for patients with or without a range of genetic defects (11). Dupilumab, which blocks IL-4 signaling to enhance Type 1 T cell responses, is effective for patients that exhibit a Th2-skewed phenotype (10). The success of these approaches emphasizes the importance of further investigation into immunologic deficits underlying anemic T cell responses in DCM to identify new therapeutic targets. PD-1 blockade has shown promising results in case reports of invasive mold infections, and may have broader applications in treatment-refractory or severe infectious diseases (34–38). Our results suggests that immune checkpoint inhibitor therapy may be a valuable therapeutic approach for some individuals with DCM and deserves further investigation.

## Methods

### Sex as a biological variable.

Individuals were recruited independently of sex, and sex was analyzed as an independent variable.

### Patient recruitment and demographics.

Patients were confirmed to have coccidioidomycosis by an infectious disease specialist from the Valley Fever Institute, University of California, Davis, or UCLA. Disease category (1) was assigned by these experts after review of diagnostic testing and clinical trajectory. Individuals were then enrolled in an IRB-approved protocol and whole blood was collected and shipped to UCLA for analysis. Demographic data were provided by patients at time of recruitment. SIRE was recorded separately as ethnicity (Hispanic or non-Hispanic) and race (Black, White, Asian, Pacific islander or Hawaiian, or American Indian) per 1997 US census guidelines. Age, sex, and date of *Coccidioides* diagnosis were similarly self-reported by individuals at recruitment.

### Arthroconidia (FKC) generation, harvest, and fixation.

*C*. *immitis* RS was obtained from BEI (RS NR-48942). Arthroconidia was harvested as previously described (95). Briefly, loopful samples from frozen stocks were inoculated onto 100 mL of 2× Glucose Yeast Extract (2× GYE; 2% glucose, Sigma-Aldrich, G8270-5KG; 1% yeast extract, Gibco DF210929) 1.5% agar (Gibco, DF0145-17-0) in T225 tissue culture flasks with 100× penicillin/streptomycin (pen/strep; 10,000 U/mL penicillin and 10,000 μg/mL streptomycin, Gibco, 15140122) and grown for 4–10 weeks at room temperature. Harvested arthroconidia were stored at 4°C. Arthroconidia viability was assessed by plating 100 μL of serially diluted samples on 2× GYE agar with pen/strep. Colony forming units (CFUs) were obtained by enumerating colonies after 3–4 days of incubation at 30°C. Fixed arthroconidia samples were generated by mixing arthroconidia aliquots with 37% formaldehyde at a 1:5 ratio (6% final formaldehyde concentration) and incubating at room temperature for at least 30 minutes. Fixed arthroconidia were washed with PBS twice, resuspended in PBS (Gibco), and stored at 4°C.

### Spherule (FKS) growth and fixation.

To generate *C*. *immitis* RS spherules, 1 × 10^6^ arthroconidia per mL were inoculated into polypropylene vented shaker flasks (Thermo Fisher Scientific, BBV12-5) containing Converse medium (96) and grown under shaking conditions (150 rpm) at 39°C supplemented with 10% CO_2_. After 5 days, mature spherules were observed. Spherules were fixed in a 1:5 ratio with 37% formaldehyde and incubated at room temperature for at least 30 minutes. The fixed spherules were washed with PBS twice, resuspended in PBS, and stored at 4°C. Spherules were quantified by direct counting using Kova hemocytometers (Thermo Fisher Scientific, 22-270141).

### Cell culture.

Cells were cultured in complete T cell medium (RPMI 1640 supplemented with 10% FCS, 10 mM HEPES, 1 mM sodium pyruvate, 55 μM 2-mercaptoethanol, and 1× Pen-Strep) at 37°C.

For AIM assays, cryopreserved PBMCs were thawed at 37°C and washed twice with T cell medium. Cells were either cultured in media alone or stimulated with FKS (4,000 spherules per 1 million cells), FKC (1,000 conidia per 1 million cells), or anti–CD3-CD2-CD28 (StemCell ImmunoCult, 10970) for 24 or 48 hours before analysis by flow cytometry. MHC-I blockade was performed with 10 μg/mL anti–HLA-A, B, C clone W6/32 (BioLegend, 311427). MHC-II blockade was performed with 10 μg/mL each of anti–HLA-DR, DP, DQ clone Tü39 (BD, 555556), and anti–HLA-DR clone L243 (BioLegend, 307665). Monoclonal antibodies for cell culture were low endotoxin and azide free. FKS-Sup referenced in Figure 2E were generated by 48-hour coculture of PBMCs with FKS, after which cells were pelleted and supernatant was collected. For this experiment, only FKS-Sup from AIM-positive individuals was used to stimulate healthy control PBMCs.

For intracellular cytokine staining, PBMCs were thawed and washed as above, and stimulated with FKS and FKC for 48 hours as above. Brefeldin A (BioLegend, 420601) was added for the final 12–24 hours of culture. Where indicated, 10 μg/mL anti–PD-1 (BioXCell, SIM0003) or IgG4 isotype control (BioXCell, CP147) was added.

### Flow cytometry.

For phenotyping, fresh or frozen PBMCs were washed with FACS buffer (PBS with 2% FCS, 1 mM EDTA), stained with a cocktail of fluorescently conjugated monoclonal antibodies for 20 minutes at 4°C, and then washed twice with FACS buffer prior to data acquisition. Cultured cells from AIM assays were stained by the same protocol. Intracellular cytokine staining samples were fixed with 2% paraformaldehyde in PBS prior to surface staining, then permeabilized with Perm Buffer (BioLegend, 421002). Intracellular cytokine staining was performed in Perm Buffer at 4°C for 35 minutes. Cells were then washed twice with Perm Buffer and twice with FACS buffer before data acquisition.

The staining panels were as follows. T cell phenotype: CD3 BV605 (BioLegend, 317322), CD4 PerCPCy5.5 (BioLegend, 350008), CD8 PE (BioLegend, 301051), CD45RA BV421 (BioLegend, 304130), CCR7 PECy7 (BioLegend, 353414), CD57 BV785 (BioLegend, 393330), PD-1 AF647 (BioLegend, 329910), HLADR AF488 (BioLegend, 327010). AIM assay: CD3 BV605, CD4 PerCPCy5.5, CD8 PE, CD69 AF488 (BioLegend, 310916), CD134 APC (BioLegend, 309818), CD137 PECy7 (BioLegend, 309818). Intracellular cytokine staining: CD3 BV605, CD4 PerCPCy5.5, CD8 PECy7 (BioLegend, 344712), IFNγ PE (BioLegend, 502509), IL-17 AF647 (BioLegend, 512310), IL-4 BV421 (BioLegend, 500826). Data were collected on a Cytek DxP10 digital flow cytometer and analyzed with FlowJo version 10.

### Statistics.

For flow cytometry results, AIM gates were drawn for each experiment using the unstimulated negative controls and anti-CD3–stimulated positive controls as guides. AIM-positive values for each assay were determined by subtracting unstimulated background from the FKC- or FKS-stimulated cells within each individual. Samples with less than 2,000 viable CD4^+^ T cells upon data acquisition were discarded. We set a cutoff value of 0.05% for background-subtracted values to be determined as positive: values below this were considered negative. Over the course of this study, AIM assays were performed with FKS or FKC for 24- or 48-hour intervals due to time, reagent, and sample constraints. We compiled results into a composite AIM score that reports the average of positive results from assays (FKS or FKC stimulation for 24 to 48 hours) performed for that individual. We determined that 24- or 48-hour stimulation with FKC or 48-hour stimulation with FKS was sufficient to detect *Coccidioides*-specific T cell responses and gave concordant results, but 24-hour FKS stimulation was not (Figure 2). Individuals with only a negative 24-hour FKS result were therefore not included.

Categorical variables were analyzed by χ^2^ or Fisher’s exact test. T cell phenotype results were assessed for normal distribution by visual inspection and analyzed by 2-tailed nonparametric tests (Wilcoxon/Mann-Whitney *U* test). Variation of phenotypic markers by age were assessed by simple linear regressions. AIM and intracellular cytokine staining assay results were analyzed by paired or unpaired nonparametric Wilcoxon tests. Correlations were analyzed by nonparametric Spearman tests. Odds ratio confidence intervals were calculated by the Baptista-Pike method. Corrections for multiple tests were not performed. Statistical analyses were performed in R version 4.5.1 (https://cran.r-project.org/bin/windows/base/old/4.5.1/) and GraphPad Prism version 10.

### Study approval.

All patients provided informed consent to participate in protocols approved by the Institutional Review Board (IRB) of UCLA, with reliance agreements from the Valley Fever Institute and the University of California, Davis.

### Data availability.

Values for all data points in graphs are reported in the Supporting Data Values file.

## Author contributions

Conceptualization: GDW, TJT, MGL, and MJB. Investigation: GDW, TJT, AVS, and MAML. Resources (fungal antigens): MMT and SB. Resources (patient and samples): GRT and RHJ. Visualization: GDW. Supervision: MJB. Funding acquisition: MJB. Writing (original): GDW. Writing (editing): All.

## Conflict of interest

The authors have declared that no conflict of interest exists.

## Funding support

This work is the result of NIH funding, in whole or in part, and is subject to the NIH Public Access Policy. Through acceptance of this federal funding, the NIH has been given a right to make the work publicly available in PubMed Central.

- National Institute of Allergy and Infectious Diseases (NIAID/NIH) grants U19 AI166059 and R21 AI149654 (to MJB).
- University of California Office of the President (UCOP) grant VFR-19-633386 (to MJB).

## Supplementary Material

Supplemental data

ICMJE disclosure forms

Supporting data values

## Figures and Tables

**Table 1:**
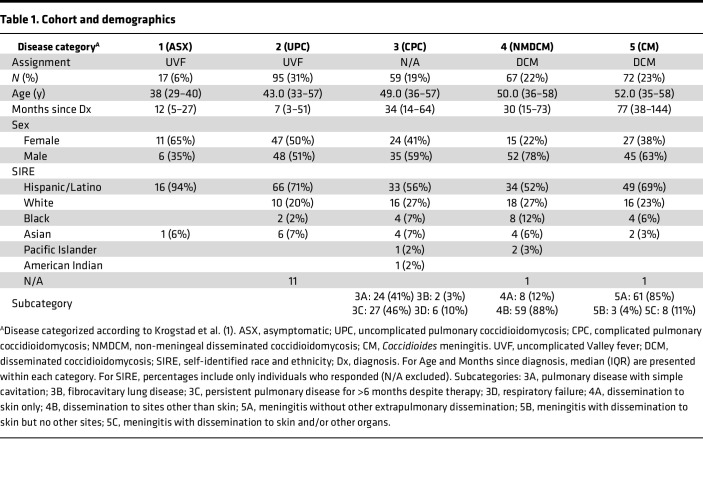
Cohort and demographics

## Version 1 04/20/2026

In-Press Preview

## Version 2 05/22/2026

Electronic publication
