# Supplementary material for: Exhausted T cell phenotypes in disseminated coccidioidomycosis
Source: JCI Insight. 2026 Apr 20;11(10):e203270. doi: 10.1172/jci.insight.203270 (PMC13232714; doi:10.1172/jci.insight.203270)
Supplement: Supplemental data [file jciinsight-11-203270-s075.pdf]

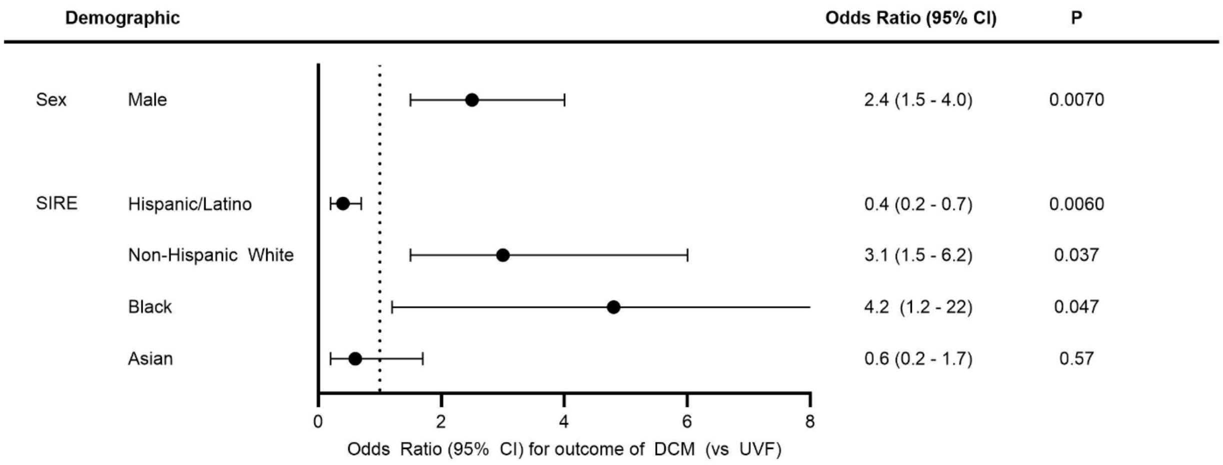

Figure S1: Forest plot showing association of sex and SIRE (self-identified race and ethnicity) with disease severity

Odds ratios of Male sex (relative to female sex) and SIRE of Hispanic (relative to non-hispanic), Non-Hispanic White (relative to non-white), black (relative to non-block), and asian (relative to non-asian) for outcome of DCM vs UVF. Odds ratios by Baptista-pike, p-values by Fisher Exact test.

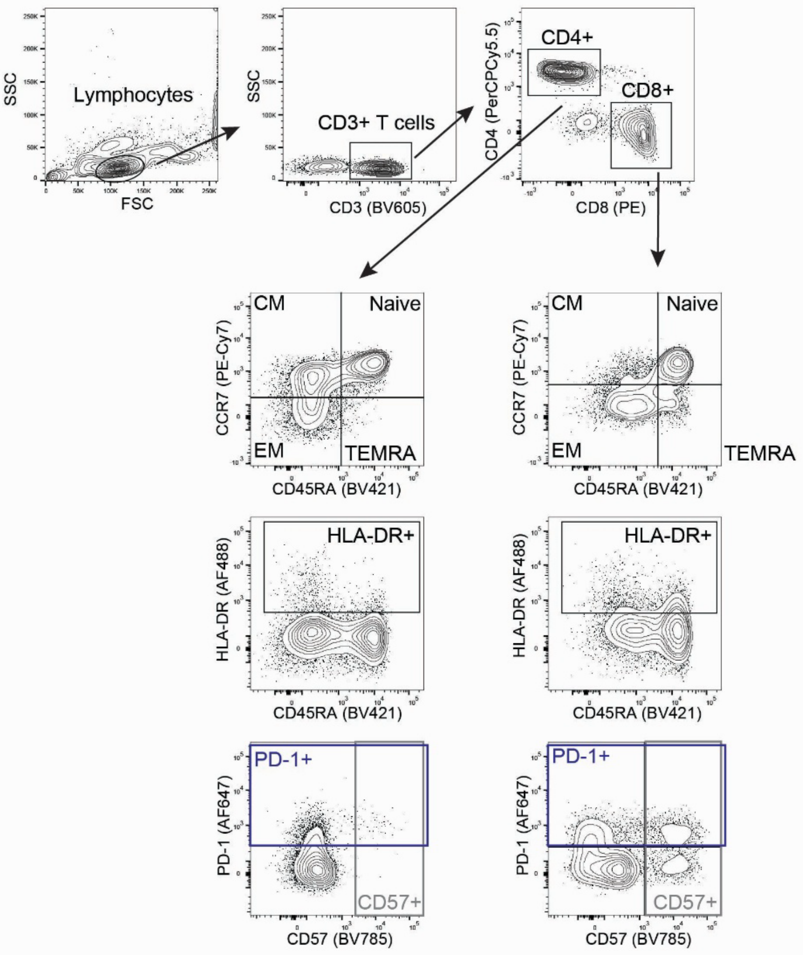

Figure S2: Representative PBMC T cell Phenotype Gating Strategy (related to **Figure 1**)

Flow cytometry staining of cryopreserved PBMCs was performed as described in methods. CM, central memory; EM, effector memory.

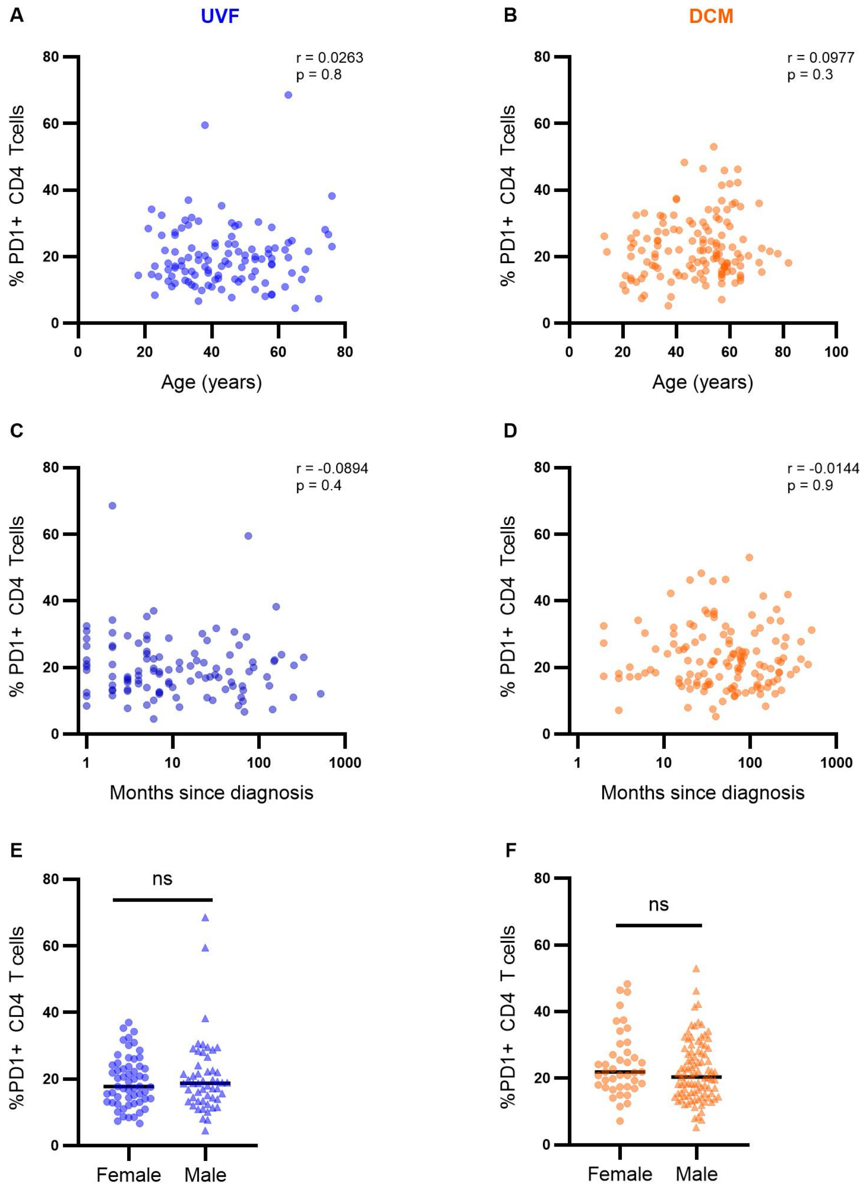

Figure S3: Association of CD4 T cell PD-1 expression with age, time since diagnosis, and sex in UVF (blue, N = 109**) and DCM (**orange**,** N = 135**)**, (related to **Figure 1**)

(**A**, **B**) Spearman correlation of %PD1+ CD4 T cells with age. (**C**, **D**) Spearman correlation of %PD1+ CD4 T cells with time since diagnosis in months. (**E**, **F**) Mann-Whitney test comparing %PD1+ CD4 T cells by sex (Female and Male). ns, not significant

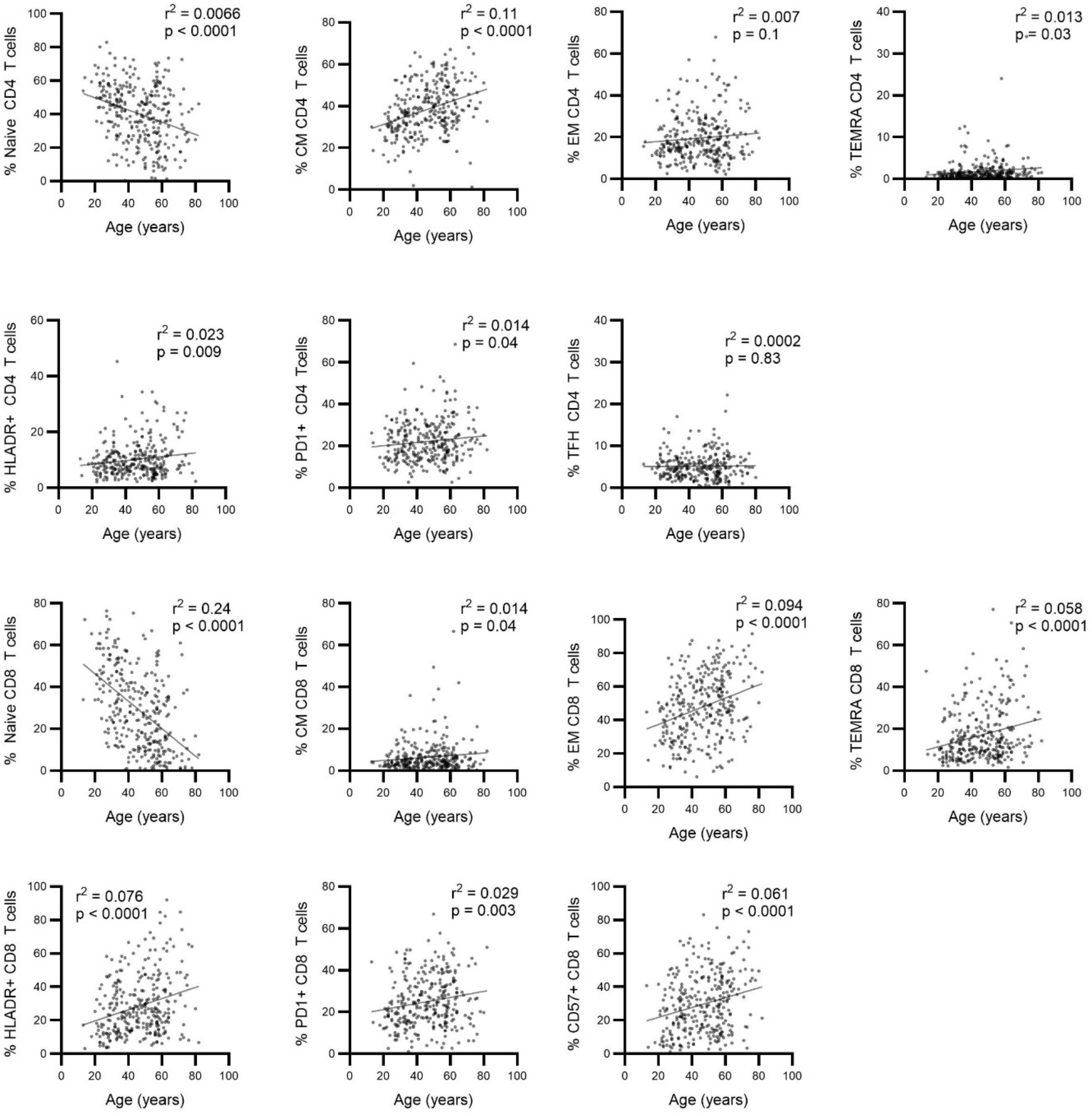

Figure S4: Variance of phenotypic markers with age (related to **Figure 1**)

Simple linear regression was performed for each phenotyped population on CD4 and CD8 T cells by subject age, r^2 and unadjusted p values are presented. N = 302 subjects.

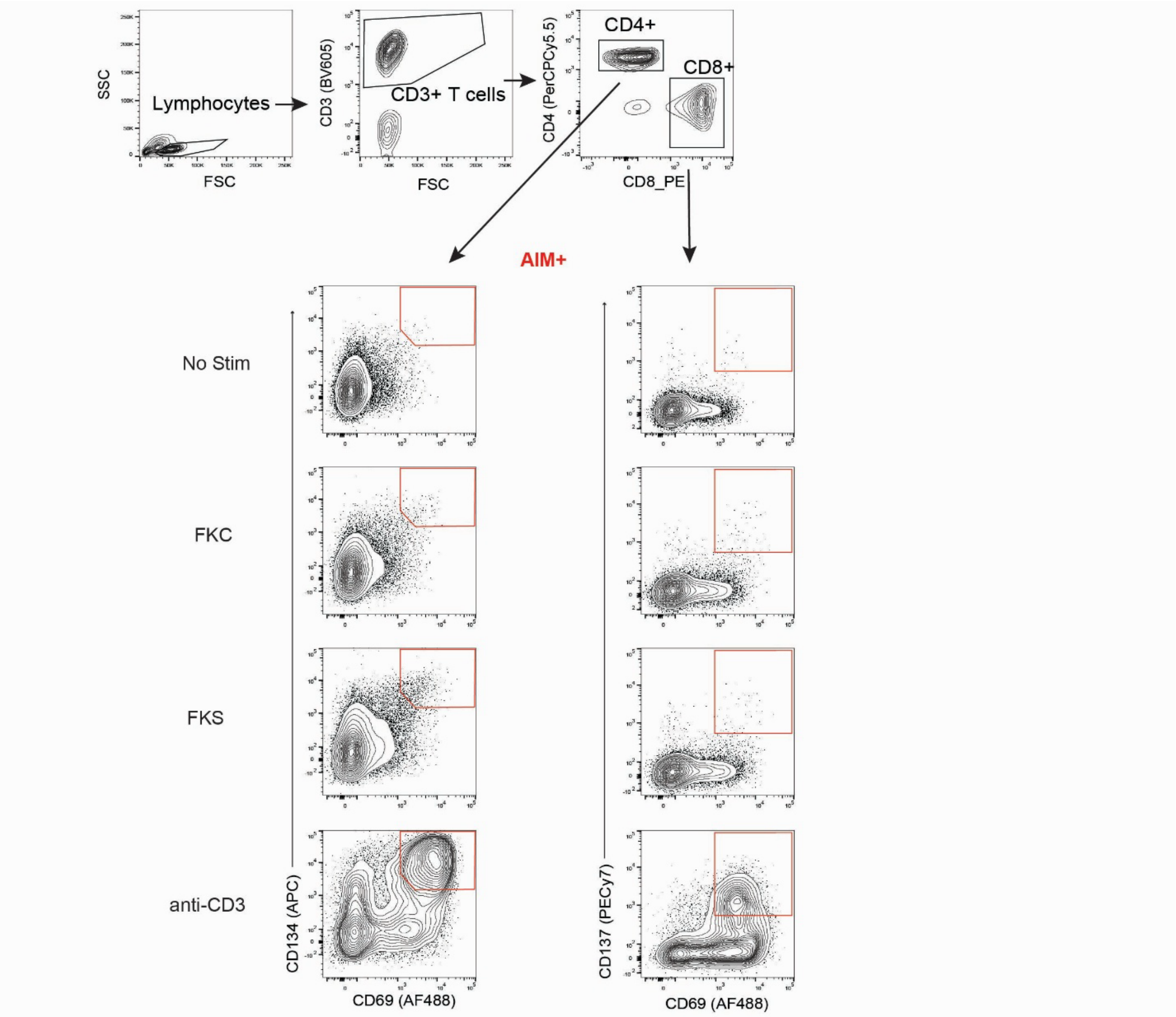

Figure S5: Representative AIM Gating Strategy (related to **Figure 2)**

Flow cytometry staining of cultured PBMCs performed as described in methods. AIM positive population (red) was determined as the CD69+CD134+ subset of CD4 T cells and CD69+CD137+ subset of CD8 T cells following culture of PBMCs alone (No Stim) or stimulation with FKC, FKS, or anti-CD3. The AIM positive population was gated conservatively to minimize background

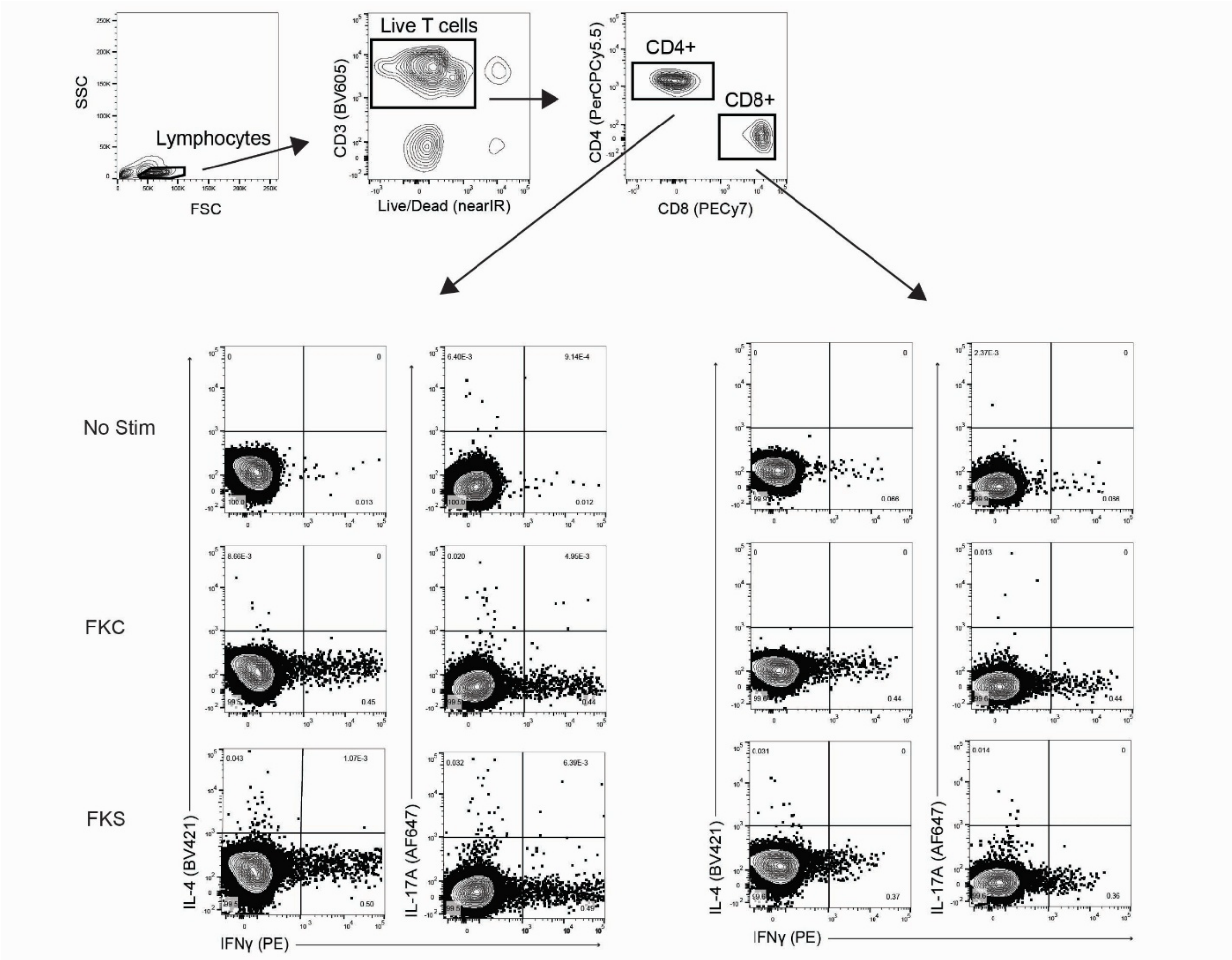

Figure S6: Representative ICS Gating Strategy (related to **Figure 3, 4)**

Intracellular staining of cultured cells was performed as described in methods. Representative gating of IFNγ, IL4, and IL17A positive cells by quadrants for CD4 and CD8 T cells is shown. Only CD4 T cell results were included in analysis as CD8 T cell responses were MHC-I-independent and may represent bystander activation (as presented in Figure 2).

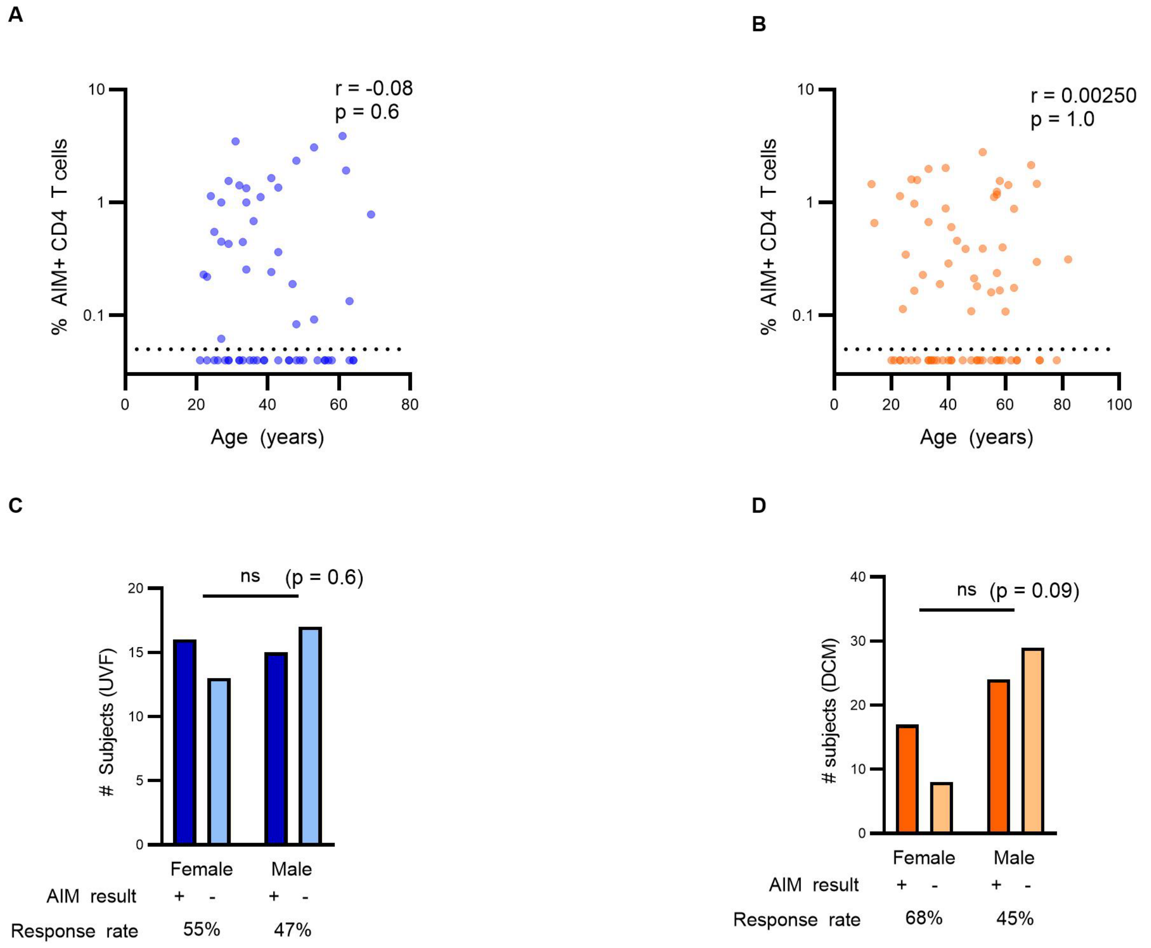

Figure S7: Association of AIM results with Age and Sex (related to **Figure 3)**

(**A**, **B**) Spearman correlation of age with %AIM+ CD4 T cells for (**A**) UVF (N = 31) and (**B**) DCM (N = 40). (**C**, **D**) Fisher exact tests comparing AIM results (positive/+ or negative/-) among male and female subjects within (**C**) UVD and (**D**) DCM. ns, not significant, $p > 0.05$.

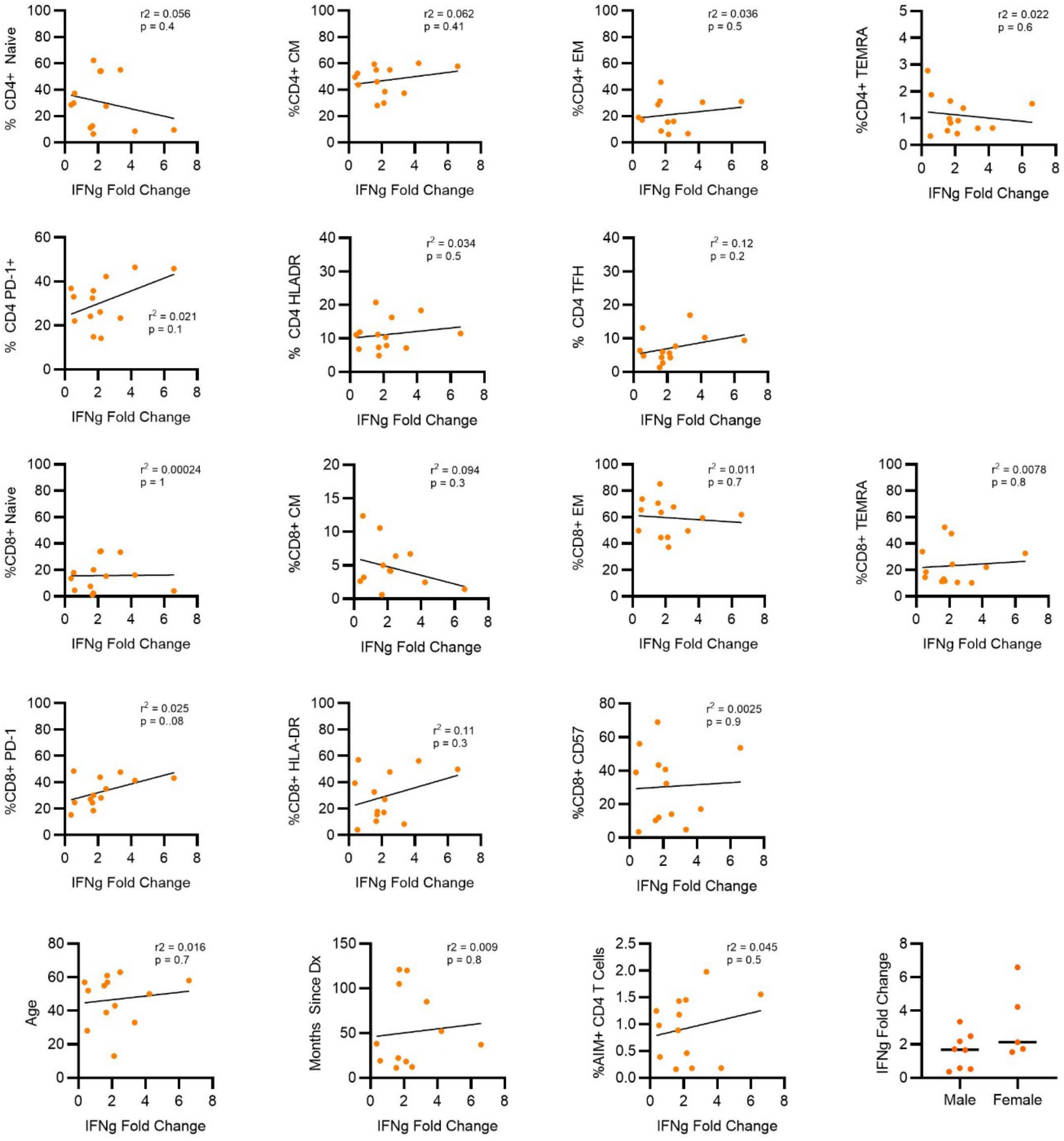

Figure S8: Variance of response to checkpoint blockade with T cell phenotypes and demographic variables

Simple linear regression was performed for fold change interferon gamma production in response to Coccidioides antigen stimulation (fold change of IFNγ) compared to T cell phenotypic markers and demographics. r^2 and unadjusted p values are presented. Variation by sex compared by Mann Whitney U test. No significant variance was found. N = 13 subjects.
